# Effect of sub-pixel multiplexing on the display quality of LED display

**DOI:** 10.1038/s41598-023-43900-6

**Published:** 2023-10-03

**Authors:** Junchang Chen, Xifeng Zheng, Yu Chen, Yang Wang, Fengxia Liu, Deju Huang, Yufeng Chen, Xinyue Mao, Hui Cao, Fanyang Xing

**Affiliations:** 1 Changchun Institute of Optics, Fine Mechanics and Physics, Chinese Academy of Science, Changchun, 130033 China; 2 University of Chinese Academy of Sciences, Beijing, 100049 China; 3 Changchun Cedar Electronics Technology Co., Ltd., Changchun, 130103 China

**Keywords:** Electrical and electronic engineering, Electronics, photonics and device physics

## Abstract

In this paper, a theoretical model is presented for the display process of uniformly-arranged virtual-pixel LED displays with RGBG sub-pixel structure cells. Such displays' modulation transfer function (MTF) is derived theoretically from this model. Experiments were conducted to validate the theoretical model to measure the MTF of virtual-pixel displays and traditional real-pixel displays with a pixel pitch of 0.9 mm. A dual-line spread function measurement method is proposed, which is experimentally shown to be more effective than the conventional single-line LSF measurement method in measuring the MTF of LED displays. The rationality of the theoretical model was analyzed and compared through experiments. Furthermore, a combined subjective and objective evaluation method for the image quality of LED sub-pixel displays is proposed, which analyses the effect of LED sub-pixel multiplexing on the display clarity based on the square root integration method and achieves the subjective goal of quantifying the LED display quality. The research results reveal the theoretical and experimental aspects of virtual-pixel displays and may have practical significance for the design of high-quality LED displays.

## Introduction

LED displays have become a popular choice in various applications due to their advantages such as high brightness and high contrast ratio. However, with the increasing demand for large-area ultra-high-definition displays, the limitations of LED chip characteristics and manufacturing processes have made it challenging to achieve high pixel density compared to LCD and OLED displays. This limitation has significantly restricted the use of LED displays in indoor scenes and consumer electronics. Consequently, there is a growing need to explore techniques to enhance the perceived resolution of LED displays with limited physical resolution, a widely researched area in the field of LED display technology.

**Figure 1:**
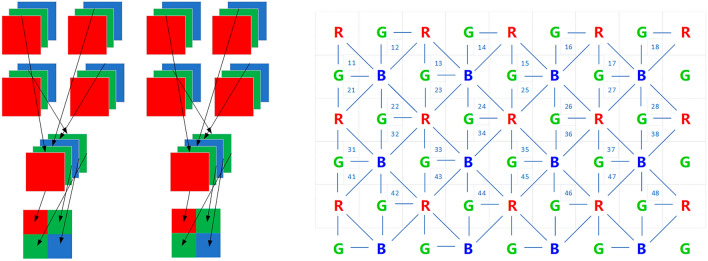
Schematic diagrams of the sub-pixel sampling algorithm (left) and sub-pixel multiplexing algorithm (right) for RGBG uniformly arranged.

Sub-pixel multiplexing and sub-pixel sampling are widely studied methods for achieving higher perceived resolution in displays. While sub-pixel sampling has been shown to have the ability to improve luminance spatial resolution, it suffers from significant colour error problems^1^, especially when displaying high-frequency signals. In contrast, sub-pixel multiplexing involves the process of smoothing the image signal followed by sub-pixel sampling. The core idea behind sub-pixel multiplexing is to achieve a perceptual resolution that exceeds the physical resolution by reusing each physical sub-pixel with several adjacent virtual pixels. An example of sub-pixel multiplexing is shown in the right-hand image in Fig. 1.

The virtual-pixel display shown in Fig. 1 utilizing sub-pixel multiplexing has a uniform sub-pixel arrangement that differs from the classic RGB stripe arrangement. In this display, the same physical sub-pixel is shared by two or four virtual pixels surrounding it, achieving the same resolution as the RGB stripe arrangement with only 1/3 of the number of LED chips. However, although this method can display more information without increasing the physical pixel resolution, there are doubts about its impact on improving image quality^2^.

X. Zhao compared and analyzed various common sub-pixel layouts according to the basic principles of chromatics and the central wavelength specifications of LED chips^2^. Klompenhouwer M. A. analyzed the display spectrum of a sub-pixel display using image signal processing to compare the display quality of a sub-pixel display and a pixel display^3^. A. Gago-Calderón developed and used a subjective method for evaluating the image quality of various types of LED displays^4^. The exclusive use of the objective analysis method, without taking into account subjective factors, may lead to a deviation from real-life viewing scenarios^5^. Conversely, a subjective evaluation method without the incorporation of objective factors may produce discrete results and require significant human effort.

Except for resolution, the Modulation Transfer Function (MTF) is a more comprehensive measure of display clarity^6^. The MTF characterizes the spatial imaging performance of an imaging system and defines the sinusoidal response of the system or its components presented to the observer^7^. The MTF of a display effectively expresses the modulation of the image content by the display and quantitatively characterizes the sharpness of the display. This study delves into the effect of sub-pixel arrangement and multiplexing algorithm on the sharpness of LED displays through an analysis of the MTF of the display, and proposes an evaluation method that combines an objective display modulation transfer function with a subjective contrast sensitivity function of the human eye. This method will form a more rigorous and reliable evaluation process and means.

## Theoretical

The sub-pixel multiplexing display encompasses a series of steps, namely sampling, virtualization, phase-shift addressing, and reconstruction^3^. We will meticulously construct a comprehensive signal domain model of sub-pixel multiplexing display, incorporating the arrangement and multiplexing algorithm depicted in Fig. 1 (right).

### Samplings

Consider a continuous image $$\left[ {\begin{array}{*{20}c} {\begin{array}{*{20}c} {R\left( {x, y} \right)} \\ {G\left( {x, y} \right)} \\ {B\left( {x, y} \right)} \\ \end{array} } \\ \end{array} } \right]$$ which shares the same dimensions as the LED display area. The process of sampling entails taking values in the spatial domain at specific horizontal and vertical intervals $\left( {p_{x} ,p_{y} } \right)$. As shown mathematically in the spatial domain by using Eq. (1).

1 $$\left[ {\begin{array}{*{20}c} {\begin{array}{*{20}c} {R\left( {m,n} \right)} \\ {G\left( {m,n} \right)} \\ {B\left( {m, n} \right)} \\ \end{array} } \\ \end{array} } \right]{ } = { }\left[ {\begin{array}{*{20}c} {\begin{array}{*{20}c} {R\left( {x, y} \right)} \\ {G\left( {x, y} \right)} \\ {B\left( {x, y} \right)} \\ \end{array} } \\ \end{array} } \right] \cdot \sum \delta \left( {x - mp_{x} , y - np_{y} } \right)$$

In this context, $$\left[ {\begin{array}{*{20}c} {\begin{array}{*{20}c} {R\left( {m,n} \right)} \\ {G\left( {m,n} \right)} \\ {B\left( {m, n} \right)} \\ \end{array} } \\ \end{array} } \right]$$ denotes the expression for the sampled discrete image resulting from the sampling process, and $p_{x}$ and $p_{y}$ correspond to the horizontal and vertical sampling intervals, respectively.

To distinguish between the continuous and discrete domains, we denote the frequency of the discrete domain as $\left( {u,v} \right)$, while the frequency of the continuous domain is represented as $\left( {f_{x} ,f_{y} } \right)$. The relationship between the two in the sampling discretization process is given by $u = p_{x} f_{x} ,v = p_{y} f_{y}$. In the frequency domain, the sampling process can be described mathematically by Eq. (2).

2 $$\left[ {\begin{array}{*{20}c} {\begin{array}{*{20}c} {R\left( {u,v} \right)} \\ {G\left( {u,v} \right)} \\ {B\left( {u,v} \right)} \\ \end{array} } \\ \end{array} } \right] = \frac{1}{{p_{x} p_{y} }} \cdot \left[ {\begin{array}{*{20}c} {\begin{array}{*{20}c} {R\left( {f_{x} ,f_{y} } \right)} \\ {G\left( {f_{x} ,f_{y} } \right)} \\ {B\left( {f_{x} ,f_{y} } \right)} \\ \end{array} } \\ \end{array} } \right]*\sum \delta \left( {f_{x} - k\frac{1}{{p_{x} }}, y - l\frac{1}{{p_{y} }}} \right)$$

where $$f_{x} = \frac{u}{{p_{x} }}$$ and $$f_{y} = \frac{v}{{p_{y} }}$$.

### The process of multiplexing

**Figure 2:**
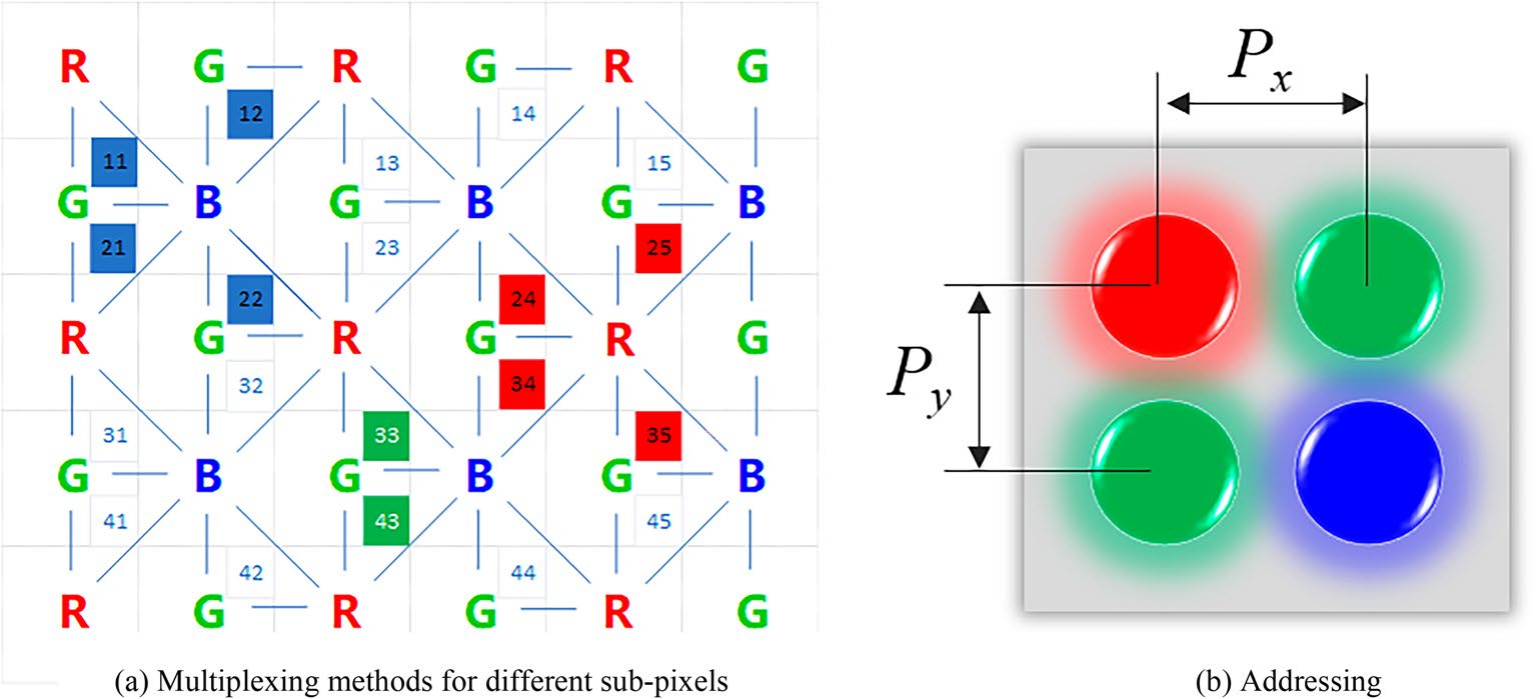
Multiplexing.

When using the RGBG quad-chip arrangement, the multiplexing process can be split into two stages. The first stage involves employing smoothing filtering in the multiplexing process, as shown in Fig. 2a.

Each small square in Fig. 2a corresponds to a pixel point used for sampling, with every 4 sub-pixels of RGBG forming a pixel structure cell. At each sampling point, the luminance of red, green, and blue is sampled simultaneously. In the traditional real-pixel display, sampling is followed by direct addressing. However, in the virtual-pixel display, multiplexing exists, so a smoothing filtering step is necessary. Since the red and blue sub-pixels are multiplexed in a four-nearest-neighbour way, smoothing filtering can be done by convolving the image with a 2 × 2 matrix whose elements are 1/4. On the other hand, the green sub-pixel has twice as many sub-pixels as the other two sub-pixels and is located at different positions in each pixel structure cell. To account for this, the sampled green sub-images are divided into two, resulting in four sub-images: R, G, B, and G sub-images. In the pixel structure cell, the two physical green sub-pixels are multiplexed as the average of the green luminance values of the two virtual pixels to their right. In essence, smoothing filtering is like multiplying the whole image by a 2 × 2 matrix. The matrix has two elements on the left side that are 1/2 and two elements on the right side that are 0. This process can be expressed as Eq. (3) in the spatial domain using mathematics.

3 $$\left[ {\begin{array}{*{20}c} {\begin{array}{*{20}c} {\overline{R}\left( {m,n} \right)} \\ {\overline{G}\left( {m,n} \right)} \\ {\overline{G}\left( {m,n} \right)} \\ {\overline{B}\left( {m, n} \right)} \\ \end{array} } \\ \end{array} } \right] = \left[ {\begin{array}{*{20}c} {\begin{array}{*{20}c} {R\left( {m,n} \right)*\frac{1}{4}\left[ {\begin{array}{*{20}c} 1 & 1 \\ 1 & 1 \\ \end{array} } \right]} \\ {G\left( {m,n} \right)*\frac{1}{2}\left[ {\begin{array}{*{20}c} 1 & 0 \\ 1 & 0 \\ \end{array} } \right]} \\ {G\left( {m,n} \right)*\frac{1}{2}\left[ {\begin{array}{*{20}c} 1 & 0 \\ 1 & 0 \\ \end{array} } \right]} \\ {B\left( {m, n} \right)*\frac{1}{4}\left[ {\begin{array}{*{20}c} 1 & 1 \\ 1 & 1 \\ \end{array} } \right]} \\ \end{array} } \\ \end{array} } \right]$$

where the sub-images $\overline{R}\left( {m,n} \right), \overline{G}\left( {m,n} \right), \overline{B}\left( {m, n} \right)$ represent the convolution process outcomes.

The results of the convolution process described above can be represented in the frequency domain as follows.

4 $$\left[ {\begin{array}{*{20}c} {\begin{array}{*{20}c} {R\left( {u,v} \right) \times e^{{ - j3\pi \left( {u + v} \right)}} cos\pi u cos\pi v} \\ {G\left( {u,v} \right) \times e^{{ - j\left( {2\pi u + 3\pi v} \right)}} cos\pi v} \\ {G\left( {u,v} \right) \times e^{{ - j\left( {2\pi u + 3\pi v} \right)}} cos\pi v} \\ {B\left( {u,v} \right) \times e^{{ - j3\pi \left( {u + v} \right)}} cos\pi u cos\pi v} \\ \end{array} } \\ \end{array} } \right]$$

After completing the convolution process, the next step in multiplexing is to down-sample the sub-images, the four sub-images of the whole image are down-sampled at half the sampling frequency in both the horizontal and vertical directions. This process is represented by Eq. (5) in the spatial domain.

5 $$\left[ {\begin{array}{*{20}c} {\begin{array}{*{20}c} {R_{O} \left( {m,n} \right)} \\ {G_{O}^{1} \left( {m,n} \right)} \\ {G_{O}^{2} \left( {m,n} \right)} \\ {B_{O} \left( {m,n} \right)} \\ \end{array} } \\ \end{array} } \right] = \left[ {\begin{array}{*{20}c} {\begin{array}{*{20}c} {\overline{R}\left( {2m - 1,2n - 1} \right)} \\ {\overline{G}\left( {2m,2n - 1} \right)} \\ {\overline{G}\left( {2m - 1,2n} \right)} \\ {\overline{B}\left( {2m,2n} \right)} \\ \end{array} } \\ \end{array} } \right]$$

In Eq. (5), $G_{O}^{1} \left( {m,n} \right)$ and $G_{O}^{2} \left( {m,n} \right)$ are the green sub-pixels located in the lower left and upper right corners, respectively, of the RGBG pixel structure cell. This process causes the sampled pixels to contain only the primary colour information, thus changing the spectral characteristics of each channel after the multiplexing process, as shown follows.

6 $$\left[ {\begin{array}{*{20}c} {\begin{array}{*{20}c} {R\left( {\frac{u}{2},\frac{v}{2}} \right) \times e^{{ - j\frac{5}{2}\pi \left( {u + v} \right)}} cos\pi \frac{u}{2} cos\pi \frac{v}{2}} \\ {G\left( {\frac{u}{2},\frac{v}{2}} \right) \times e^{{ - j\left( {\pi u + \frac{5}{2}\pi v} \right)}} cos\pi \frac{v}{2}} \\ {G\left( {\frac{u}{2},\frac{v}{2}} \right) \times e^{{ - j\left( {2\pi u + \frac{3}{2}\pi v} \right)}} cos\pi \frac{v}{2}} \\ {B\left( {\frac{u}{2},\frac{v}{2}} \right) \times e^{{ - j\frac{3}{2}\pi \left( {u + v} \right)}} cos\pi \frac{u}{2} cos\pi \frac{v}{2}} \\ \end{array} } \\ \end{array} } \right]$$

Notice that in the derivation between Eq. (5) and Eq. (6), the Fourier expression for the down-sampling process is not actually directly realisable in terms of the telescoping frequency axis, since the expression for the spectral transformation of the down-sampling process should be: $X_{d} \left( {e^{j2\pi u} } \right) = \frac{1}{M}\mathop \sum \limits_{i = 1}^{M} X\left( {e^{{j\left( {2\pi u/M - 2\pi i/M} \right)}} } \right)$ Where $X_{d} \left( {e^{j2\pi u} } \right)$ is the discrete signal spectrum after down-sampling, $X\left( {e^{j2\pi u} } \right)$ is the discrete signal spectrum before down-sampling, and $M$ is the sampling interval for down-sampling. The $i/M$ is the aliasing term, and when the down-sampling frequency is not high enough, i.e. the down-sampling interval $M$ is not small enough, the aliasing term will affect the shape of the spectrum after down-sampling, but here, since an averaging algorithm of smoothing filter is done before down-sampling, this filter can be regarded as an anti-aliasing filter, so after this anti-aliasing filter, the aliasing induced by the $i/M$ term can be ignored.

### The process of addressing

The discrete images are subjected to a reconstruction process that converts them into a continuous image for subsequent analysis, where spatial domain reconstruction involves converting discrete digital coordinates into continuous sampling point coordinates.

In the multiplexing process, the down-sampling doubles the pixel pitch. This is because each small square in Fig. 2a is a sampling pixel point, and after pixel multiplexing, each RGBG pixel structure cell is considered a complete pixel point, thereby resulting in a pixel structure pitch that is twice that of the real-pixel display unit, i.e., $\left( {2p_{x} ,2p_{y} } \right)$, as is shown in Fig. 2b. Consequently, the mapping relationship from the discrete domain frequency to the continuous domain frequency during the reconstruction process is $u = 2p_{x} f_{x} , v = 2p_{y} f_{y}$. As a result, the spectrum of the image is modified to:

7 $$\left[ {\begin{array}{*{20}c} {\begin{array}{*{20}c} {R\left( {f_{x} ,f_{y} } \right) \times e^{{ - j5\pi \left( {p_{x} f_{x} + p_{y} f_{y} } \right)}} cos\pi p_{x} f_{x} cos\pi p_{y} f_{y} } \\ {G\left( {f_{x} ,f_{y} } \right) \times e^{{ - j\pi \left( {2p_{x} f_{x} + 5p_{y} f_{y} } \right)}} cos\pi p_{y} f_{y} } \\ {G\left( {f_{x} ,f_{y} } \right) \times e^{{ - j\pi \left( {4p_{x} f_{x} + 3p_{y} f_{y} } \right)}} cos\pi p_{y} f_{y} } \\ {B\left( {f_{x} ,f_{y} } \right) \times e^{{ - j3\pi \left( {p_{x} f_{x} + p_{y} f_{y} } \right)}} cos\pi p_{x} f_{x} cos\pi v} \\ \end{array} } \\ \end{array} } \right]$$

The process of LED display after reconstruction starts with addressing:

The RGBG chips of a sub-pixel multiplexing display are uniformly arranged and require convolution with a colour offset matrix during addressing. In the frequency domain, it is only necessary to multiply the spectral functions by a phase shift matrix.

8 $$\left[ {\begin{array}{*{20}c} {e^{{ - j\pi \left( {p_{x} f_{x} + p_{y} f_{y} } \right)}} } & 0 & 0 & 0 \\ 0 & {e^{{ - j\pi \left( { - p_{x} f_{x} + p_{y} f_{y} } \right)}} } & 0 & 0 \\ 0 & 0 & {e^{{ - j\pi \left( {p_{x} f_{x} - p_{y} f_{y} } \right)}} } & 0 \\ 0 & 0 & 0 & {e^{{ - j\pi \left( { - p_{x} f_{x} - p_{y} f_{y} } \right)}} } \\ \end{array} } \right]$$

In the following step, the light diffusion of each LED chip in the display is modelled as a point spread function. To facilitate analysis, this function is approximated as a two-dimensional rectangle function with dimensions of $\frac{{p_{x} }}{2}$ and $\frac{{p_{y} }}{2}$, The spectrum of this rectangle function is a sampling function $\frac{{sin\pi p_{x} f_{x} }}{{\pi p_{x} f_{x} }} \cdot \frac{{sin\pi p_{y} f_{y} }}{{\pi p_{y} f_{y} }}$.

The subsequent step in LED virtual-pixel display after reconstruction involves convolving the continuous and addressed image with the point spread function, which in the frequency domain is achieved by multiplying each sub-pixel channel spectrum by the sampling function.

With the completion of the above-mentioned sampling, multiplexing, and addressing processes, the spectral representation of the ultimate displayed image can be expressed by Eq. (9).

9 $$\left[ {\begin{array}{*{20}l} {R_{c} \left( {f_{x} ,f_{y} } \right) \times e^{{ - j6\pi \left( {p_{x} f_{x} + p_{y} f_{y} } \right)}} \frac{{sin2\pi p_{x} f_{x} }}{{2\pi p_{x} f_{x} }} \cdot \frac{{sin2\pi p_{y} f_{y} }}{{2\pi p_{y} f_{y} }}} \hfill \\ {G_{c} \left( {f_{x} ,f_{y} } \right) \times e^{{ - j\pi \left( {p_{x} f_{x} + 6p_{y} f_{y} } \right)}} \frac{{sin\pi p_{x} f_{x} }}{{\pi p_{x} f_{x} }} \cdot \frac{{sin2\pi p_{y} f_{y} }}{{2\pi p_{y} f_{y} }}} \hfill \\ {G_{c} \left( {f_{x} ,f_{y} } \right) \times e^{{ - j\pi \left( {5p_{x} f_{x} + 2p_{y} f_{y} } \right)}} \frac{{sin\pi p_{x} f_{x} }}{{\pi p_{x} f_{x} }} \cdot \frac{{sin2\pi p_{y} f_{y} }}{{2\pi p_{y} f_{y} }}} \hfill \\ {B_{c} \left( {f_{x} ,f_{y} } \right) \times e^{{ - j2\pi \left( {p_{x} f_{x} + p_{y} f_{y} } \right)}} \frac{{sin2\pi p_{x} f_{x} }}{{2\pi p_{x} f_{x} }} \cdot \frac{{sin2\pi p_{y} f_{y} }}{{2\pi p_{y} f_{y} }}} \hfill \\ \end{array} } \right]$$

As above, we have established a mathematical model for the sub-pixel multiplexing display process based on the RGBG quad-chip arrangement, which includes matrix components on the left $R_{c} \left( {f_{x} ,f_{y} } \right)$, $G_{c} \left( {f_{x} ,f_{y} } \right)$, and $B_{c} \left( {f_{x} ,f_{y} } \right)$ representing the monochromatic spectrum functions of the original continuous image. The right-hand side of the matrix comprises the product of the phase shift factor and the $sinc$ function, which represents the transfer function of the entire display system. The model indicates that the virtual display process involves amplitude low-pass filtering and phase shifting of the source image, with the former attributed to the multiplexing and reconstruction processes, while the latter arises from the multiplexing and phase offset addressing processes. The product of the two $sinc$ functions, obtained by taking the modulus of the right part of the spectrum other than the source image, is the monochromatic MTF expected theoretically.

## Experimental

In order to validate the accuracy and validity of the MTF model proposed in the preceding section, a series of experiments were conducted to measure the horizontal MTF (This is because the human eye has a better ability to discriminate horizontally than vertically) of displays with RGBG sub-pixel uniform arrangement and RGB vertical stripe arrangement. In order to eliminate the potential effects of extraneous variables, we selected two LED displays with a pixel pitch of 0.9 mm arranged in the two ways mentioned above for measurement. A high-resolution CMOS industrial camera was used as the measuring device (MD). The camera has a resolution of 65 million pixels (9344 × 7000). The measurements are taken as follows.

### Measuring the MTF of the measurement device

**Figure 3:**
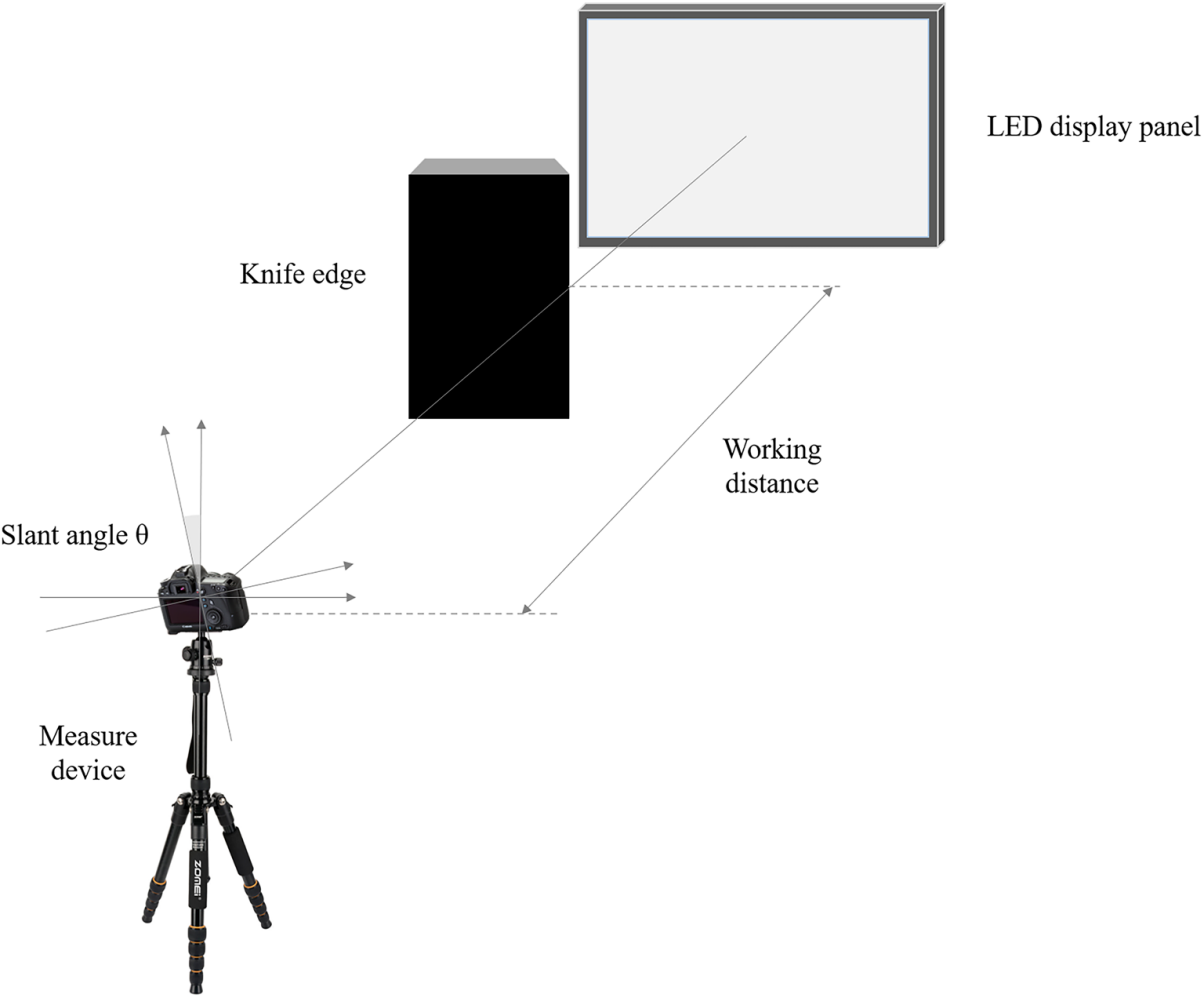
Schematic of the MTF measurement for the measurement device.

To minimize the influence of the MD on the subsequent MTF assessment and to ensure that the results are reliable and accurate, it's necessary to measure the MTF of the MD, which is determined using an edge-based method under controlled lighting conditions in a dark room^8^. The setup for the MD MTF measurement is illustrated in Fig. 3.

A. A black object with straight and knife edges was placed parallel to the display and the MD, which was positioned at a distance of 1600 mm from the object. The foreground object and the background display were separated by a distance of 600 mm. The MD was focused on the black object in the centre with its optical axis perpendicular to the display and the black object. The display showed pure colour patches of red, green, blue, and white. The MD was rotated around the optical axis by a certain angle $\theta$ so that the edge of the black object was tilted with respect to the sampling array of the measurement device, which was calculated in the next step.
B. For this experiment, 5 images were captured for LED display panels with both arrangements. To reduce noise, the captured images were denoised using the averaging method. Next, the images are rotated by an angle $\theta$ to maintain a vertical edge, and then a small square area containing the edge of the image is selected as the region of interest (ROI) for the experiment.
  The ROI image is averaged along the vertical direction to generate a one-dimensional horizontal edge spread function.
C. A one-dimensional line spread function (LSF) is produced by applying a one-dimensional difference filter $$\left[\begin{array}{ccc}-1& 0& 1\end{array}\right]$$.
D. A TUKEY window is applied to the LSF, which is then Fourier transformed to obtain the Optical Transfer Function (OTF). The amplitude of the OTF yields the MTF of the MD, which is subsequently normalized by the sum of all pixel grey values. The units of the frequency axis are in terms of ${f}_{MD}$ at this stage.

**Figure 4:**
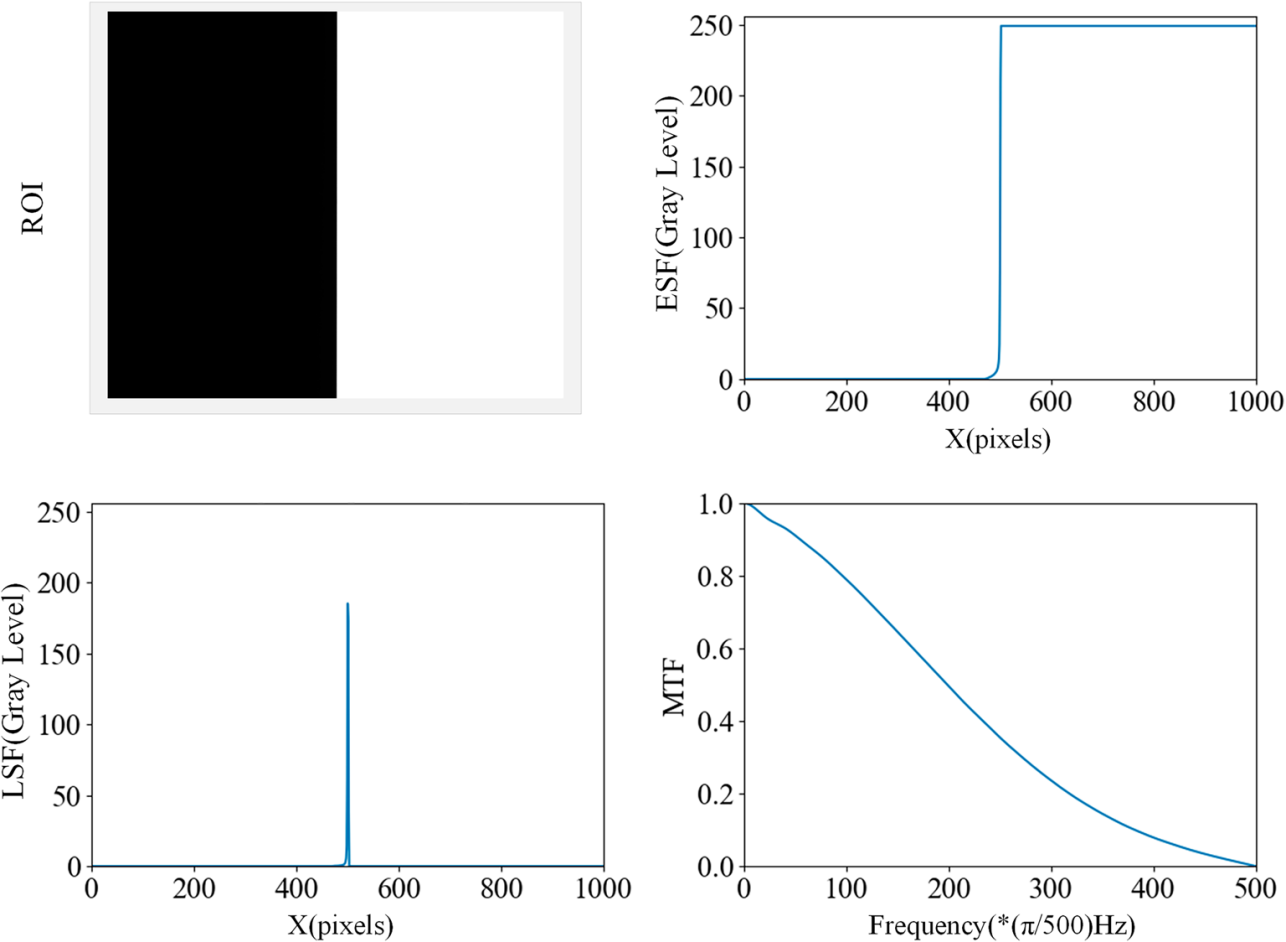
MTF measuring of the measurement device.

The measurement results are shown in Fig. 4, the ROI of the captured edge image has a resolution of 1000 × 1000 pixels, so the one-dimensional LSF data consists of 1000 points, and after performing the FFT on the LSF, 1000 points of DFT are obtained, the first 500 points can be used to analyze the MTF of the MD, which gradually decreases in the interval [0, 500] (i.e., the full frequency band [0,$\pi$] in the continuous frequency domain of the discrete signal) until it reaches 0 at the highest frequency of 500.

### Measuring the pixel ratio

To determine the pixel ratio (m) between the MD and the LED display, an image with a black background and a white circle with a radius of 100 pixels in the foreground is generated by a computer and displayed on the LED display to be measured. The camera is positioned in line with the previous step, and an image of the circle is captured by the MD. Least squares fitting is performed to obtain the pixel ratio. The result shows that the pixel ratio $m$ is 10.38.

### Measuring the MTF of the whole system

A. Under unchanged camera position and angle, a vertical straight line with single and double pixel widths of red, green, blue, and white is displayed on the LED display panel with two different arrangement^9^. For each scene, 5 images are captured with the MD.
B. The resulting images are first denoised, and then a least-squares fitting is performed on the original image with respect to the tilt angle^10^, where the fitted equation is represented by Eq. (10).
  10 $$v\left( {x,y} \right) = e^{{\frac{{ - \left( {x - x_{0} - tan\theta \cdot y} \right)^{2} }}{{2\sigma^{2} }}}}$$
  The result of the fit yields its tilt angle $\theta$ as -9.779185492°.
C. The noise-reduced image is rotated clockwise by $\theta$ angle to ensure the verticality of the line to be measured. The one-dimensional LSF is then sampled directly as in the previous step. The MTF of the entire system is obtained by performing the Fourier transform after the TUKEY window processing, then taking the modulus and normalizing it.
D. Divide the modulation transfer function of the entire system $\left( {MTF_{ES} } \right)$ by the modulation transfer function of the measurement device $\left( {MTF_{MD} } \right)$ to obtain the final estimate of the modulation transfer function of the display device $\left( {MTF_{DISP} } \right)$ ^7^.

**Figure 5:**
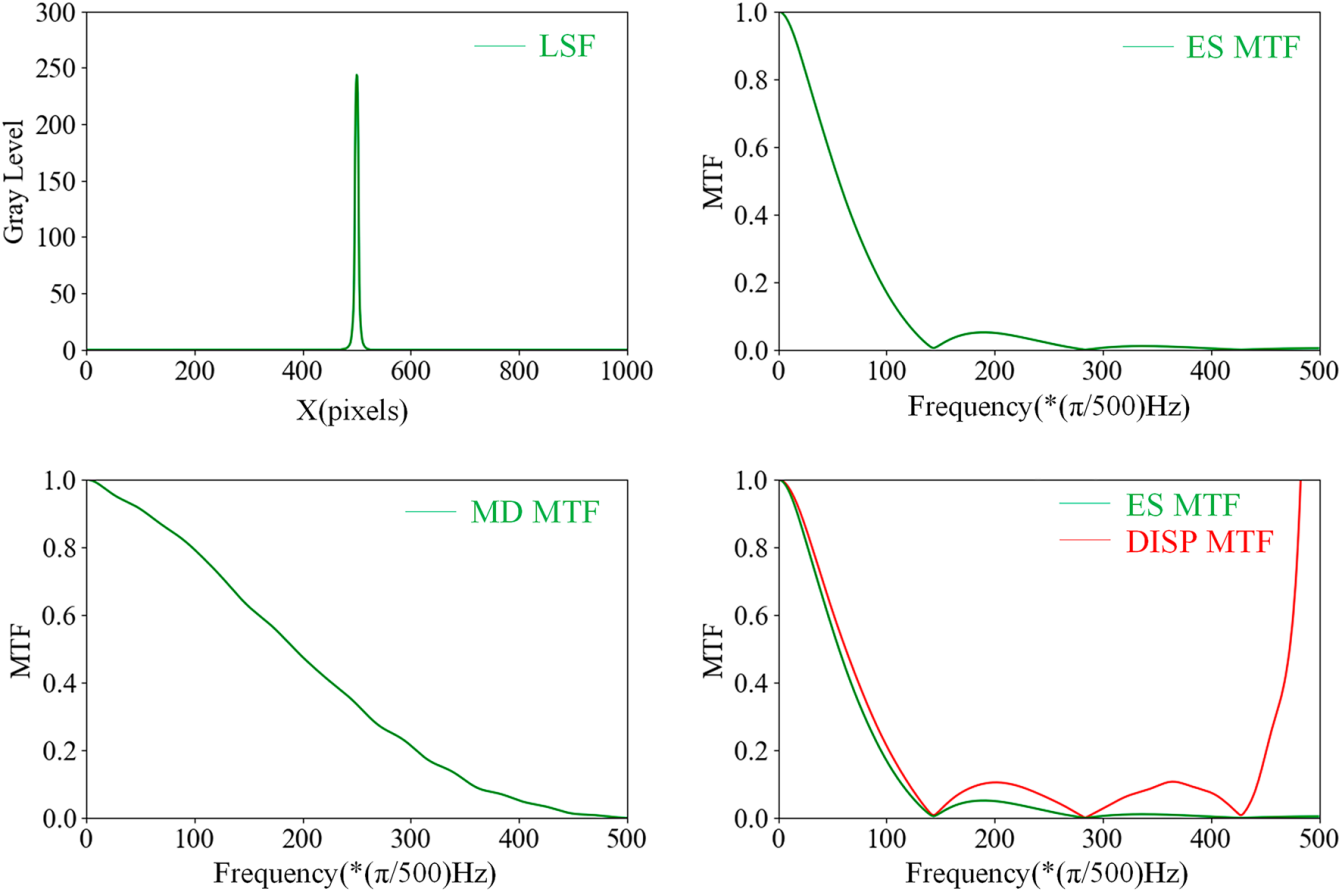
*MTF*_*ES*_, *MTF*_*MD*_, *MTF*_*DISP*_ for the first measurement.

Figure 5 shows the results of the first experimental estimation of the MTF for a real-pixel display with a pixel pitch of 0.9 mm.

### Frequency axis transformation

**Figure 6:**
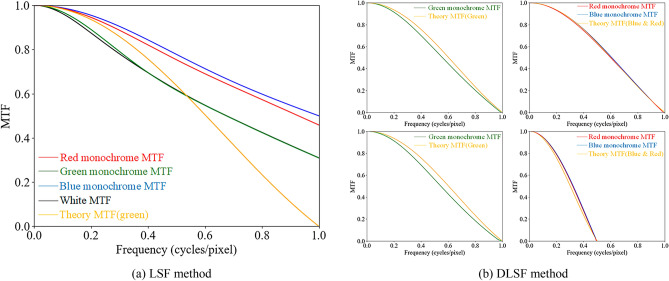
The comparison between measured and theoretical results using LSF and DLSF method.

Assuming a pixel pitch of $p$ for the display, a measured distance of $d$ between the MD and the display, and a pixel ratio of $m$ obtained in section “Measuring the pixel ratio”, the MTF obtained from the measurement (with a unit of $f_{MD}$) must be converted to the spatial frequency domain $f_{c}$ in $cycles/pixel$, in order to unify it with the frequency of the continuous spatial domain on the LED display. Specifically, $f_{c} = \frac{m}{N}f_{MD}$. With a unified frequency axis, the MTF derived from the model built in the previous section and the experimentally measured MTF of the real-pixel display can be compared in Fig. 6a.

### The DLSF method

Based on the analysis of Fig. 6a, it is clear that the MTF curve obtained by measuring vertical lines of a single pixel width does not closely align with the empirical measurements and theoretical derivation^7^. We contend that for LED displays, especially LED virtual pixel displays, the display quality is closely related to the pixel pitch. However, the traditional single-line LSF method for measuring the line spread function does not incorporate information about the pixel pitch. This is due to the fact that, irrespective of the pixel pitch, as long as the LED model and luminous information remain the same, the result will be identical when only a single line is measured. To address this issue, we introduce the DLSF method, which measures two single-pixel vertical lines that are adjacent to one another, and the physical distance between them on the display is considered to be the pixel pitch of the virtual-pixel LED display. Consequently, the measurement results include pixel pitch information.

Considering the entire LED display system as a linear shift-invariant system, where the spatial domain expression of a single pixel line is represented by $\delta \left( n \right)$, and the spatial domain expression of a double pixel line is represented by $\delta \left( n \right)$ + $\delta \left( {n - 1} \right)$, the dual-line impulse response can be obtained by adding the impulse response of a single pixel line, $h\left( n \right)$ to that of a shifted line, $h\left( {n - 1} \right)$, resulting in $g\left( n \right) = h\left( n \right) + h\left( {n - 1} \right)$, upon Fourier transformation, the dual-line frequency response of the display system can be expressed as Eq. (11).

11 $$G\left( {e^{j2\pi f} } \right) = H\left( {e^{j2\pi f} } \right) + H\left( {e^{j2\pi f} } \right) \cdot e^{ - j2\pi f} = 2 \cdot H\left( {e^{j2\pi f} } \right) \cdot e^{ - j\pi f} \cdot cos\pi f$$

According to Kim^6^ and Infante^11^, the resulting modulation transfer function (MTF) for a real-pixel display is a two-dimensional sampling function given by $\frac{{sin\pi p_{x} f_{x} }}{{\pi p_{x} f_{x} }} \cdot \frac{{sin\pi p_{y} f_{y} }}{{\pi p_{y} f_{y} }}$. For the digital image, the pixel pitch loses its effect, so for the x-direction, the MTF becomes $\frac{{sin\pi f_{x} }}{{\pi f_{x} }}$. Additionally, for the dual-line frequency response, we have:

12 $$G\left( {e^{{j2\pi f_{x} }} } \right) = 2 \cdot \frac{{sin\pi f_{x} }}{{\pi f_{x} }} \cdot e^{{ - j\pi f_{x} }} \cdot cos\pi f_{x} = 2 \cdot \frac{{sin2\pi f_{x} }}{{2\pi f_{x} }} \cdot e^{{ - j\pi f_{x} }}$$

As the amplitude factor 2 and the phase factor $e^{{ - j\pi f_{x} }}$ lose their influence during the MTF calculation process due to the normalization and modulus steps, to accurately measure the MTF using the DLSF method, it is only necessary to stretch the frequency axis by a factor of 2 after completing all the calculation steps of the single-line LSF method.

Since the part of the frequency that exceeds the cutoff frequency is already a non-modulable frequency for the display system, it is necessary to remove the frequency components that exceed the cutoff frequency to compare the measured and model-derived MTFs. This can be achieved by erasing the corresponding part of the MTF curve.

Finally, the horizontal MTF curves for both virtual-pixel and real-pixel displays obtained from the DLSF method measurement assessment are shown in Fig. 6b.

In Fig. 6b, the top two plots are for the real-pixel display and the bottom two plots are for the virtual-pixel display. The red, green, and blue curves represent the monochromatic MTFs of the corresponding colours, and the orange curve represents the horizontal MTFs derived from the model in Section Theoretical.

The modulation cut-off frequency of the real-pixel display system is $1 cycles/pixel$ which is a fundamental limit due to the inability to display more than one cycle of information with a single pixel point. This limit applies to all three primary colours and the mixed colour. On the other hand, in the virtual-pixel display, the R and B sub-pixels are multiplexed in both the $x$ and $y$ directions, leading to a reduction in their MTF cutoff frequency in the $x$ direction to $0.5 cycles/pixel$, As a result, the resolution of the R and B sub-pixels in the $x$-direction is only half that of the conventional real-pixel display. In contrast, the multiplexing of the G sub-pixels is only performed in the $y$-direction, resulting in the MTF cut-off frequency and modulation depth of the G sub-pixel in the $x$-direction being identical to those of the conventional real-pixel display.

The MTF curves shown in Fig. 6b demonstrate that using the DLSF method produces results that match the theoretical derivation outlined in Section Theoretical. Additionally, these curves are superior to those obtained using the single-line LSF method. This confirms the validity of the theoretical derivation in Section Theoretical and indicates that the DLSF method is better suited for measuring the MTF of LED displays. Consequently, data collected from the DLSF method will be used in subsequent analyses instead of the single-line LSF method.

### Fitting of the luminance MTF

To analyze the luminance modulation transfer function of a display system, the RGB colour gamut can be converted to the YUV colour gamut model. The conversion formula is as follows:

13 $$\left[ {\begin{array}{*{20}c} Y \\ U \\ V \\ \end{array} } \right] = \left[ {\begin{array}{*{20}c} {0.30} & {0.59} & {0.11} \\ { - 0.17} & { - 0.33} & {0.50} \\ {0.50} & { - 0.42} & { - 0.08} \\ \end{array} } \right] \cdot \left[ {\begin{array}{*{20}c} R \\ G \\ B \\ \end{array} } \right]$$

The luminance MTF of the virtual-pixel display can be analyzed in terms of the luminance component Y, which is calculated as a linear combination of the luminance of the red (R), green (G), and blue (B) sub-pixels, as $Y = 0.3R + 0.59G + 0.11B$. The MTF of the R and B sub-pixels follow a $\frac{sin2\pi f}{{2\pi f}}$ response, while that of the G sub-pixel follows a $\frac{sin\pi f}{{\pi f}}$ response. Therefore, the overall luminance MTF can be obtained as a weighted average of the MTFs of the individual sub-pixels, using the appropriate weights.

14 $$0.41 \cdot \frac{sin2\pi f}{{2\pi f}} + 0.59 \cdot \frac{sin\pi f}{{\pi f}}$$

**Figure 7:**
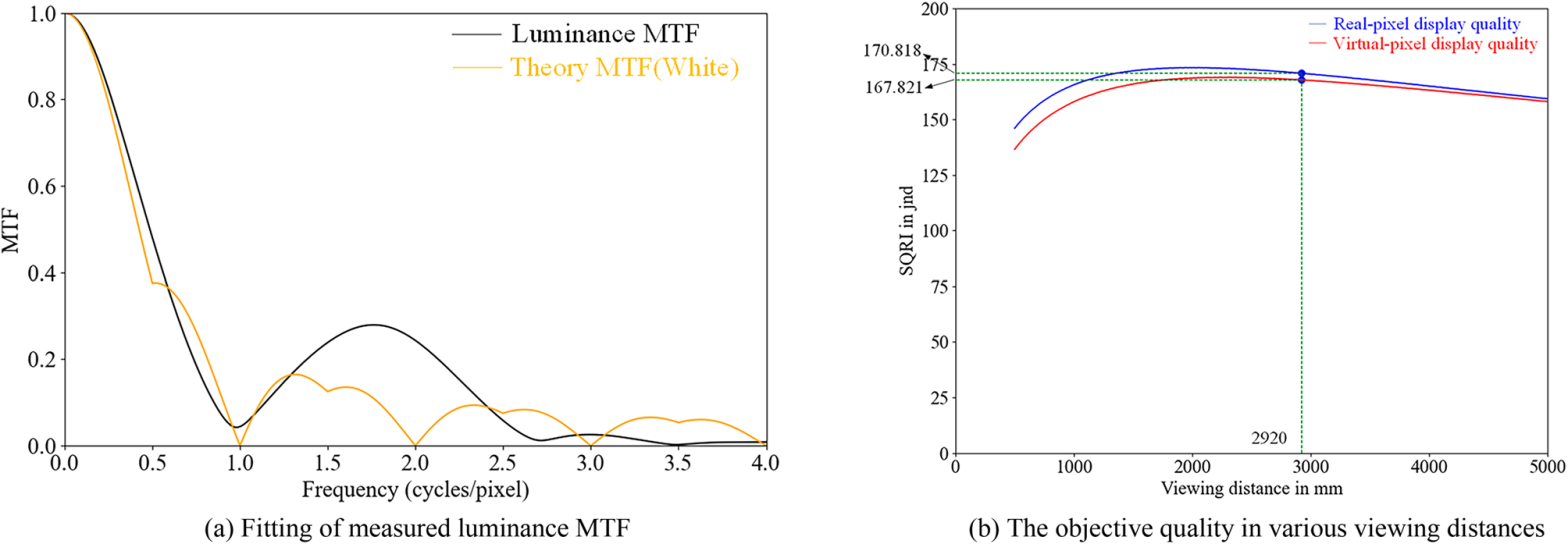
Fitting of luminance MTF and objective quality in various distances.

It has been shown that Eq. (14) provides an adequate fit to the measured data, which is presented in Fig. 7a.

Based on the above analysis, it can be concluded that in a virtual-pixel display with an RGBG four-chip arrangement, the presence of the extra green sub-pixel in the RGBG pixel structure cell increases the cut-off frequency of the luminance MTF curve in the *x*-direction, as observed in the partial frequency range between *0.5 cycles/pixel* and *1 cycle/pixel* in Fig. 7a. This effect exceeds the modulation capability achievable with a conventional real-pixel LED display which would require three times the number of sub-pixels. Thus, the pixel-multiplexing display technology can improve the modulation capability of the display for the same number of pixels.

## Results

The Contrast Sensitivity Function (CSF) gives contrast sensitivity as a function of spatial frequency. In the CSF, the spatial frequency is expressed in, that is, the number of display pattern cycles per degree of human visual angle^12^. It reaches a maximum between 1 and 10 cycles per degree with a fall off at higher and lower spatial frequencies^13,14^. Barten has formulated the contrast sensitivity function with a physical model and developed the square-root integral(SQRI) method to evaluate the subjective quality of the displayed image in conjunction with the modulation transfer function of the display^15^.

Barten's theoretical model is based on a threshold function $m_{t} \left( u \right)$ limited by the internal noise of the visual system, its inverse, according to Barten, is the CSF with the spatial frequency $u$ in $cycles/degree$ ^13^. For a standard observer and usual observation conditions^16^, the function can be given by Eq. (15).

15 $$CSF\left( u \right) = \frac{{5200e^{{ - 0.0016u^{2} \left( {1 + 100/L} \right)^{0.08} }} }}{{\sqrt {\left( {1 + \frac{144}{{X_{0}^{2} }} + 0.64u^{2} } \right)\left( {\frac{63}{{L^{0.83} }} + \frac{1}{{1 - e^{{ - 0.02u^{2} }} }}} \right)} }}$$

In this equation, $u$ is the spatial frequency in $cycles/degree$, $L$ is the luminance in $cd/m^{2}$, and $X_{0}^{2}$ is the angular object area in square degrees.

The SQRI gives a good agreement with human perception of image quality, which is given by Eq. (16).

16 $$SQRI = \frac{1}{ln2}\mathop \smallint \limits_{0}^{{u_{max} }} \sqrt {\frac{MTF\left( u \right)}{{m_{t} \left( u \right)}}\frac{du}{u}}$$

where the SQRI is specified in terms of the number of perceptible distinction thresholds in a unit of $jnd$ (just noticeable differences). It should be noted that an increase of $1 jnd$ must be considered practically insignificant, an increase of $3 jnd^{\prime}s$ as significant, and an increase of $10 jnd^{\prime}s$ as substantial^15,17^.

Using the SQRI method with the display MTF model of this paper and Barten's CSF model, we can quantitatively evaluate the subjective displayed image quality of the virtual-pixel display of the quad-chip arrangement and the real-pixel display.

Note that the SQRI method requires a spatial frequency of $cycles/degree$, so we need to convert the unit of the luminance MTF to $cycles/degree$.

17 $$MTF\left( u \right) = 0.59 \cdot \frac{sin180pu/d}{{180pu/d}} + 0.41 \cdot \frac{sin360pu/d}{{360pu/d}}$$

In Eq. (17), $u = \frac{\pi d}{{180p}}f$, is the spatial frequency in $cycles/degree$, $f$ is the spatial frequency in $cycles/pixel$, $d$ is the viewing distance between the observer and the display, and $p$ is the pixel pitch of the display in millimetres.

Using the SQRI method, we calculate the subjective quality of the images on the LED real-pixel display and the virtual-pixel display at different viewing distances and plot the images in Fig. 7b (The resolution of both monitors is 1920 × 1080, and the luminance of these displays is 600 nits, the angle of measurement is perpendicular to the screen plane and right at the centre of the screen. From these parameters and the pixel pitch and viewing distance, the angular object area can be calculated).

As can be seen from Fig. 7b, when the viewing distance exceeds 2920 mm, which is approximately the optimal viewing distance for LED displays with a pixel pitch of 0.9 mm, the subjective image quality difference between the LED virtual-pixel display and the real-pixel display is less than $3 jnd$, which is insignificant, while when the viewing distance exceeds 5000 mm, the objective image quality difference between the LED virtual-pixel display and the real-pixel display is less than $1 jnd$, which is completely imperceptible.

Thus, the subjective difference in quality between LED virtual-pixel displays and real-pixel displays is always present. Still, it becomes unnoticeable when the viewing distance exceeds the optimum viewing distance.

## Conclusion

The LED display industry faces a challenge of pixel density is difficult to continue to improve. The sub-pixel multiplexing method provides a solution. To analyze the role of sub-pixel multiplexing technology on the display quality, this study presents a theoretical model of the virtual display process, which leads to a modulation transfer function (MTF) model for the LED virtual display.

Furthermore, the study designs MTF measurement experiments to verify the theoretical model and proposes a DLSF measurement method. The results demonstrate that the theoretical model fits the measured data well, thereby validating the rationality of the established model.

Finally, the study analyzed the MTF curves of the virtual-pixel LED display, quantified the subjective quality of the LED display using the square root integration method, analyzed the effect of the LED pixel multiplexing display on the definition, and concluded that the sub-pixel multiplexing display technology improves the modulation capability of the display system and achieves approximately the same display effect as the real-pixel display with more pixels at a given viewing distance.

In conclusion, this paper proposes a theoretical calculation model for MTF in sub-pixel multiplexing LED displays, and verifies the model's validity through experimentation. Combined with Barten's contrast sensitivity function model of the human eye and the square-root integration method, we have designed a method to evaluate the display quality of LED displays. Our findings indicate the influence of pixel multiplexing on the display quality and offer a more reliable method for future evaluations. This study provides theoretical support for the design of virtual-pixel displays of LEDs.

## Data Availability

The datasets used and/or analysed during the current study available from the corresponding author on reasonable request.
